# Enhancement of radiation response by inhibition of Aurora-A kinase using siRNA or a selective Aurora kinase inhibitor PHA680632 in p53-deficient cancer cells

**DOI:** 10.1038/sj.bjc.6604083

**Published:** 2007-11-20

**Authors:** Y Tao, P Zhang, V Frascogna, Y Lecluse, A Auperin, J Bourhis, E Deutsch

**Affiliations:** 1 Laboratory UPRES EA27-10 Radiosensitivity of tumors and normal tissues, University Paris XI, Institut Gustave-Roussy, Villejuif, France; 2 Department of Radiation Oncology of Cancer Hospital, Fu Dan University, Shanghai, China; 3 Institut Gustave-Roussy, Villejuif, France; 4 Department of Biostatistics and Epidemiology, Institut Gustave-Roussy, Villejuif, France

**Keywords:** cell cycle checkpoints, PHA680632, Aurora-A, p53, ionising radiation

## Abstract

Overexpression of Aurora-A kinase has been correlated with cancer susceptibility and poor prognosis in several human cancers. In this study, we evaluated the effect of inhibition of Aurora-A kinase on cell cycle progression and tumour cell survival after exposure to ionising radiation (IR). Combined IR and Aurora-A inhibition by short interfering RNA (siRNA) or by PHA680632 (a selective Aurora kinase inhibitor with submicromolar activity against Aurora-A) prior to IR led to an enhancement of radiation-induced annexin V positive cells, micronuclei formation, and Brca1 foci formation only in cells with deficient p53. However, the drug brought about additive to sub-additive interaction with radiation with regard to *in vitro* clonogenic survival. Cell cycle analysis revealed a high >4*N* DNA content 24 h after PHA680632 exposure. DNA content >4*N* was reduced dramatically when cells were irradiated combined with PHA680632 simultaneously. *In vivo* xenografts (p53−/− HCT116) of a mice study showed enhanced tumour growth delay (TGD) after the PHA680632−IR combinatorial treatment compared with IR alone. These results demonstrate that PHA680632 in association with radiation leads to an additive effect in cancer cells, especially in the p53-deficient cells, but does not act as a radiosensitiser *in vitro* or *in vivo*.

The Aurora kinases constitute one family of serine/threonine kinases whose activity is essential for the mitotic progression (Nigg, 2001; Carmena and Earnshaw, 2003). Its peak expression is from phase G2 to cytokinesis. There are three types of mammalian Aurora kinases: Aurora-A, Aurora-B, and Aurora-C. Despite their similarities, the three mammalian Aurora kinases show differences in subcellular localisations, timing of activation and functions during mitosis.

Aurora-B is one of the chromosomal passenger proteins that are essential for a number of processes during mitosis. In mammalian cells, Aurora-B forms a large chromosome passenger complex with INCENP, surviving, and Borealin (Adams *et al*, 2001; Carmena and Earnshaw, 2003; Gassmann *et al*, 2004). Aurora B function is required for mitotic chromosome alignment, spindle checkpoint function, and cytokinesis. Aurora-C has been shown to localise to spindle poles in late stages of mitosis, and a recent report indicates that it is a chromosome passenger.

Aurora-A is localised to the duplicated centrosomes and to the spindle poles in mitosis. Several studies show a role of Aurora-A in several processes required for building a bipolar spindle apparatus, including centrosome maturation and separation. Aurora-A binds to, and its kinase activity is regulated by, a protein called TPX2, which is required for spindle assembly. Aurora-A recruits important components for spindle assembly. Repression of Aurora-A expression by RNA interference (RNAi) delays mitotic entry in human cells, and overexpression of the kinase can compromise spindle-checkpoint function as well as inhibit cytokinesis (Hirota *et al*, 2003). Aurora-A possesses some phosphorylation substrates such as BRCA1 (Ouchi *et al*, 2004) and CDC25B in G2/M transition (Dutertre *et al*, 2004; Cazales *et al*, 2005). Aurora-A is implicated in p53 degradation via MDM2 (Katayama *et al*, 2004), which could further contribute to genomic instability and transformation by abrogating the ability of the cell to respond to DNA damage or other insults. Cells with overexpression of Aurora-A can resist to taxol, and this overexpression can inhibit radio-induced G2–M arrest (Anand *et al*, 2003). A diminution of cisplatin-induced apoptosis has been observed in MCF-7 cells and this effect is p53-dependent (Katayama *et al*, 2004).

Amplification of Aurora genes, as well as mRNA and protein overexpression, has frequently been reported in many human cancer cell lines: colon–rectum, breast, pancreas, and ovary. Moreover, their genetic localisations (Aurora-A, 20q13; Aurora-B, 17q13) map to chromosomal loci frequently altered in tumours. Aurora-A has been shown to act as an oncogene because overexpression of wild-type Aurora-A or of a constitutive active mutant transforms Rat1 and NIH 3T3 cells leading to colony formation in soft agar assays. In addition, NIH 3T3 cells expressing constitutively active Aurora-A can grow as solid tumours when injected into nude mice. Overexpression of Aurora-A is likely to induce a low level of genetic instability, through abnormal centrosome duplication and the generation of aneuploidy. These properties make the Aurora kinases attractive targets for anticancer therapy; indeed, the first inhibitors have been tested in the clinical setting. Several Aurora kinases inhibitors (Keen and Taylor, 2004) have been described previously: ZM447439 (Ditchfield *et al*, 2003), Hesperadin (Hauf *et al*, 2003) and VX-680 (Harrington *et al*, 2004) and, more recently, AZD1152 and MLN8054 and so on. The effect of combining Aurora-A inhibition with IR is unknown, and the aim of this study was to evaluate the influence of inhibition of Aurora-A kinase of tumour radio-sensitivity by either a genetic inhibition using short interfering RNA (siRNA) targeting Aurora-A or a pharmacological approach using a selective inhibitor PHA680632 (Soncini *et al*, 2006).

## MATERIALS AND METHODS

### Cell lines

HCT116 human colorectal cancer cell lines (wild-type (wt), p53−/−) (Bunz *et al*, 1998) were a kind gift by B Vogelstein (Johns Hopkins, Baltimore) HT29 colorectal cancer cell line (p53 mutated) and A549 human non-small cell lung cancer (wild-type p53) were obtained from the American Type Culture Collection (Manassas, VA, USA). HCT116 and HT29 cells were maintained in McCoy's 5a medium (Gibco, Gergy Pouloisa, France) supplemented with 10% fetal bovine serum (ATGC), 1% PS (Gibco), 1% L-glutamine (Eurobio, Courtaboul, France), 1 mM sodium pyruvate (Gibco), and 10 mM HEPES (Sigma, Saint Louis, MO, USA) in humidified atmosphere containing 5% CO_2_ at 37°C. A549 was maintained in RPMI-1640 medium (Eurobio) supplemented with 10% fetal bovine serum (ATGC), 1% PS (Gibco), 1% L-glutamine (Eurobio), 1 mM sodium pyruvate (Gibco), and 10 mM HEPES (Sigma).

### Clonogenic survival assays

Clonogenic survival assays were studied in p53 wt and p53−/− HCT116, HT29 (p53 and ras mutant) colorectal cancer cell lines and A549 lung cancer cell line. Cells were seeded in triplicate into six-well plates or a 25-cm^2^ flask in a range of 100–80 000 cells per well, depending on the radiation dose that the cells received, the test condition, and different cell lines, so as to yield 20–200 colonies per flask or well). Once the cells were attached, a single dose of photon irradiation with and without the drugs was applied. Cells were cultured in a 37°C, 5% CO_2_ incubator for 10–14 days. Individual colonies (>50 cells per colony) were fixed and stained for 20 min with a solution containing crystal violet and methanol. The plating efficiency (PE) represents the percentage of cells seeded that grow into colonies under a specific culture condition of a given cell line. The survival fraction, expressed as a function of irradiation, was calculated as follows: Survival fraction (SF) at 2 Gy (SF2)=colonies counted of 2 Gy/(cells seeded of 2 Gy^*^PE/100). In the clonogenic survival curve, we normalised the different conditions according to the control. The radiation dose enhancement ratio (DER) by PHA680632 was calculated using the following formula: DER=(SF at an indicated dose of radiation alone)/(SF at an indicated dose of radiation+PHA680632). Radio-sensitisation is defined as the term used when PHA680632 increases the sensitivity of cells to radiation (as assessed by clonogenic inhibition or apoptosis). This is calculated as per the formula listed above and represented in the form of DER. Thus, DER is defined as the ratio of surviving cells with radiation alone compared with a combination of radiation and PHA680632 exposures. Dose enhancement ratio=1 suggests an additive radiation effect and DER>1, a supra-additive effect as against a sub-additive effect in the case of DER<1. A 200 kV X-ray device (0.66 Gy min^−1^) and 137-caesium source (1.85 Gy min^−1^) were used *in vitro*.

### Immunocytochemistry, foci, micronuclei, and antibodies

HCT116 cells were seeded in 12-well plates incubated with a different concentration of PHA680632 for 1 or 24 h and fixed in 4% paraformaldehyde (Sigma) for 30 min. Cells were then permeabilised with PBS containing 0.1% Triton X-100 for 3 min and incubated with a 5 mg ml^−1^ BSA/PBS blocking solution. Cells were incubated with Phospho-T288 Aurora-A by a rabbit polyclonal antibody (1 : 1000, Abcam, Cambridge, UK) and with *β*-tubulin by a mouse monoclonal anti-*β*-tubulin (1 : 200, Cytoskeleton, Denver, CO, USA) primary antibody, followed by incubation with a goat anti-rabbit IgG conjugated to Alexa 555 fluorochrome (Molecular Probes, Carlsbad, CA, USA) and an anti-mouse IgG Alexa 488 conjugate (1 : 500, Molecular Probes) secondary antibody. Chromosomes were stained with 1 *μ*g ml^−1^ Hoechst 33324 (Molecular Probes) in PBS for 5 min. Tri-colour images were merged using Adobe Photoshop (v 8.0). Detection of micronuclei: cells were transfected by siRNA Aurora-A or control for 24 h, IR of 6 or 0 Gy. At 24 h of incubation after IR, the cells were stained with Hoechst 33342 (Zhang *et al*, 2006). Micronuclei and multinuclei were checked by fluorescence microscopy and morphologically classified according to standard criteria (Zhang *et al*, 2006). More than 400 cells were scored for each data point. BRCA1 foci test: cells were transfected by siRNA Aurora-A or control for 24 h, IR of 6 or 0 Gy, 4 h after incubation, and cells were fixed and stained with 1 : 500 antibody anti-BRCA1 (Santa Cruz) and 1 : 500 second antibody goat anti-rabbit IgG Alexa 555. For the quantification of BRCA1 foci, we counted only the number of the clearest foci with high intensity in each cell and excluded those foci with low intensity. A mouse monoclonal antibody against Aurora-A kinase (BD Biosciences, Franklin Lakes, NJ, USA) at a 1 : 250 dilution was used for western blotting.

### Cell cycle analysis

Sham control and 6 Gy-irradiated cells with and without drug exposure were harvested by trypsinisation at the indicated time after irradiation, washed with ice-cold PBS, fixed in 70% ethanol, and stored at −20°C. Prior to DNA analysis, DNA content was labelled with 0.1 *μ*g ml^−1^ propidium iodide (PI) and 1 mg ml^−1^ RNAse. Cell cycle analysis was performed by flow cytometry analysis (FACS Calibur, BD Biosciences).

### Annexin V staining analysis in FACS

Cells were collected by centrifugation and resuspended in 500 *μ*l annexin labelling solution consisting of 5 *μ*l Annexin V FITC (BD Biosciences) and incubated in the dark for 15 min. A total of 0.1 *μ*g ml^−1^ PI was then added to the cell suspension, followed by cytofluorimetric analysis on a FACS Vantage SE (BD Biosciences) (Zhang *et al*, 2006).

### Short interference RNA

The following siRNAs were followed for analyses: human Aurora-A (Qiagen, Valencia, CA, USA), 5′-AUUCUUCCCAGCGCGUUCC-3′ (corresponding to nucleotides 155–173 relative to the start codon) or 5′-AUGCCCUGUCUUACUGUCA-3′ (nucleotides 725–743) and the data with the first have been shown (Hirota *et al*, 2003). A double-stranded RNA non-specific (Dharmacon RNA Technologies, Cafayette, CO, USA) was used as a control. For Aurora-A siRNA analysis, the oligonucleotide targeted to positions 725–743 was used unless indicated otherwise. Two siRNAs, p53 5′-GACUCCAGUGGUAAUCCACTT-3′ and 5′-GUGAGCGCUUCGAGAUGUUTT-3′ (Qiagen), have been used for the silencing of p53, and the second has been successfully confirmed in previous siRNA experiments. The siRNA transfections were conducted with Oligofectamine transfection reagent (Invitrogen, Carlsbad, CA, USA). Annealing of the component siRNA strands and transfection were performed as described previously (Elbashir *et al*, 2001).

### Chemical Aurora-A inhibitors

Inhibitor of Aurora-A kinase, PHA-680632 (Soncini *et al*, 2006) (a kind gift from Nerviano Medical Sciences, Milan, Italy), is a specific inhibitor of Aurora kinases A, B, and C (relative molecular mass: 501 Da). The stock solution (10 mM in DMSO) was aliquoted and stored at −20°C frozen until ready for use. It was shown to be a potent inhibitor of all three Aurora kinases with IC_50_ values of 27, 135, and 120 nM for Aurora-A, -B, and -C, respectively. For *in vivo* use, PHA680632 was dissolved in 20% Tween-80 in 5% glucose solution and was stable for 3 days at 4°C. It is important to note that different concentrations of various reagents were used in different cell lines because of their relative sensitivity or resistance to the reagents tested.

### *In vivo* xenograft in nude mice

Female athymic nude mice 6–8 weeks of age (Janvier CERT 53940, Le Genest St Isle, France) were used for the tumour xenograft model. The *in vivo* experiments were carried out at the Institut Gustave Roussy under the Animal Care license C94-076-11 (Ministere de l'Agriculture). A total of 3 × 10^6^ p53−/− HCT116 cells were subcutaneously inoculated in the right flank of each mouse. Treatment began when the tumour was at least 5 mm in diameter. Mice were randomly allocated into four groups (six mice per group): A, control; B, IR alone, 8 Gy in 1 day; C, PHA680632 alone, 40 mg kg^−1^, b.i.d., for 4 days; D, same dose of PHA680632 combined with IR (24 h after the first administration of PHA680632, similar schedule as IR alone) for 4 days. Drug or vehicle control (same volume of 20% Tween-80 in 5% glucose solution) was administered intraperitoneally (i.p.). The tumour size was measured twice a week using an electronic caliper. Follow-up of individual mice was conducted. The tumour volume was estimated from 2D tumour measurements using the following formula: Tumour volume=length (mm) × width^2^ (mm^2^)/2.

### Statistical analyses

For the polyploidy of cell cycle of different conditions, a two-tailed *t*-test was used to calculate the *P*-value. The effect of PHA680632 and of dose of irradiation on the percentage of cells leading to colony formation was tested by logistic regression. To avoid repeated analyses and the increase of the *α* error rate, we studied the interaction between PHA680632 and dose of irradiation. A two-sided *χ*^2^ test was used to calculate the *P*-value of micronuclei formation difference. The effects of treatments on tumour volume were assessed using mixed models analysing the tumour volume at D4, 8, 11, 15, and 18. This model allows us to analyse the effect of treatment and the interaction between treatment and time, and to analyse repeated measurements. Statistical analysis was performed using SAS version 8.02 software (SAS Institute Inc., Cary, NC, USA).

## RESULTS

### Selective Aurora kinase inhibitor, PHA680632, inhibited colony formation in different cancer cell lines and induced polyploidy

**Figure 1:**
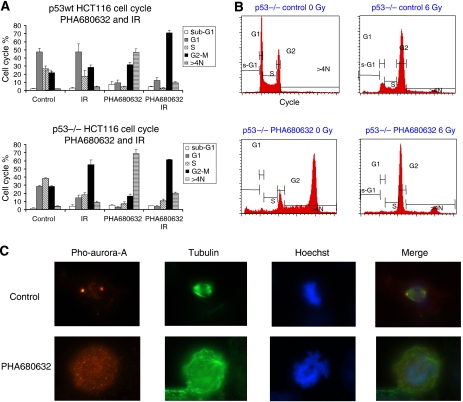
Influence of PHA680632 on cell cycle in p53wt *vs* p53−/− HCT116 cells. (**A** and **B**) analysis of the cell cycle. (**A**) Quantitative data of cell cycle distribution after PHA680632 and 6 Gy of irradiation in p53wt HCT116 (above) and p53−/− HCT116 (below) have been shown in the two histograms. The mean values (percentage of sub-population of different cell cycle: sub-G1, G1, S, G2–M, and >4*N* cells is shown in different conditions: control, IR, PHA680632, or PHA680632+IR combination) of three independent experiments are shown and bar errors represent s.e.m. Twenty-four hours exposure to 400 nM PHA680632 led to the apparition of >4*N* DNA content cells in the two HCT116 cell lines (*P*=0.0081 and *P*=0.0005 for p53wt and p53−/− HCT116, respectively), PHA680632 induced a greater accumulation of cells with >4*N* DNA content in p53−/− HCT116 cell line when compared to their p53 wild counterparts (*P*=0.0482); a moderate G2–M block was observed 24 h after 6 Gy irradiation in the control cells. At 24 h after 6 Gy irradiation and PHA680632, exposure dramatically reduced the percentage of cells with >4*N* DNA content cells compared with PHA680632 alone (*P*=0.0068 and *P*=0.0119 for p53wt and p53−/− HCT116, respectively). (**B**) A representative cell cycle analysis in p53−/− HCT116 cells. (**C**) Immunofluorescence images showing phospho-T288-Aurora-A (Pho-Aurora-A) in mitotic p53wt HCT116 cells after 24 h exposure of 1 *μ*M PHA680632 (below) or control (above). *β*-Tubulin and Hoechst were used to visualise microtubule and DNA, respectively.

We first used a selective Aurora kinase inhibitor with potential inhibition of Aurora-A, PHA680632, which has been described recently (Soncini *et al*, 2006). Polyploidy has been shown to correlate with Aurora kinases inhibition (Harrington *et al*, 2004), and we used it as a surrogate of PHA680632 efficacy on Aurora kinases. Cell cycle analysis showed that PHA680632 exposure at concentrations ranging from 200 to 400 nM induced polyploidy in HCT116 cell line with a dose dependency effect for the induction of polyploidy (data not shown). In Figure 1A, we show the percentage of different cell cycle sub-populations: sub-G1, G1, S, G2–M, and >4*N* cells after exposure to different conditions: control, IR, PHA680632 or PHA680632+IR combination. DMSO (as a control) or 400 nM PHA680632 was combined with a 6 Gy irradiation. In the two cell lines, we observe a significant increase of >4*N* cells sub-population after 24 h exposure of 400 nM PHA680632 (*P*=0.0081 and *P*=0.0005 for p53wt and p53−/− HCT116, respectively). This effect was more pronounced in p53−/− HCT116 cells than in p53wt HCT116 cells. Exposure of cells to PHA680632 for 24 h induced >4*N* DNA content in the p53−/− HCT116 cell line (69%) than in the p53 wild-type HCT116 cell line (47%), *P*=0.0482. When 6 Gy irradiation was performed after 1 h PHA680632 exposure, the >4*N* DNA content cell accumulation (>4*N* cells percentage) reduced dramatically in the p53wt HCT116 cell line (reduced to 9.6%) when compared to the same cells exposed to PHA680632 without irradiation *P*=0.0068. A similar effect has also been observed in the p53−/− HCT116 cell line (>4*N* DNA content cells reduced to 20% when 6 Gy irradiation was performed after 1 h PHA680632 exposure), *P*=0.0119.

In our study, we observed a remarkable inhibition of phosphorylation of Aurora-A in T288 in the early mitotic cells 24 h after PHA680632 treatment. Among the cells at the G2/M transition (two centrosomes), we distinguished the G2 from the M cells according to morphological criteria (condensation status of chromosomes). We also observed the G2 cells component, which is not in mitosis; these cells also present with marked phosphorylated Aurora-A in T288, while in cells treated by PHA680632, the phosphorylation of Aurora-A in T288 in these G2 cells has also been completely inhibited (data not shown). Moreover, the entire disorder of mitotic spindle in the cells treated by PHA680632 indicated a disturbed centrosome function following Aurora-A kinase inhibition (Figure 1C).

**Figure 2:**
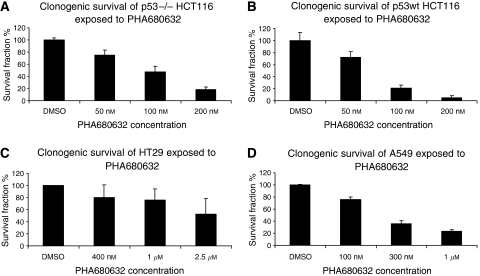
Influence of PHA680632 on clonogenic survival in different cancer cell lines. Clonogenic survival assay of cell lines exposed to a concentration range of PHA680632. (**A**) p53−/− HCT116; (**B**) p53wt HCT116; (**C**) HT29 (p53 mutated); (**D**) A549 (p53 wt).

In further clonogenic assay, PHA680632 (24 h exposure) proved to be an effective inhibitor of colony formation *in vitro*, with a dose-dependent effect at the range of 50 nM to 2.5 *μ*M in different cell lines. Clonogenic survival of HCT116, HT29, and A549 cells exposed to a concentration range of PHA680632 are shown in Figure 2. PHA680632 could inhibit the colony formation even with a concentration of 50–100 nM in HCT116 cell lines, while 1 *μ*M of PHA680632 induced only a slight clonogenic survival reduction in HT29 cells. This colony formation inhibition by PHA680632 depends on different characteristics of various cell lines and is probably dependent on the p53 or ras status of the cells. We observed that the p53−/− HCT116 is more resistant to this Aurora-A inhibitor alone than the p53 wild-type counterparts. The resistant HT29 is p53 and K-ras mutated, while the A549 with wild-type p53 is more sensitive.

### Aurora-A inhibition by PHA680632 enhanced radiation response in cancer cells, especially in p53-deficient cells

**Figure 3:**
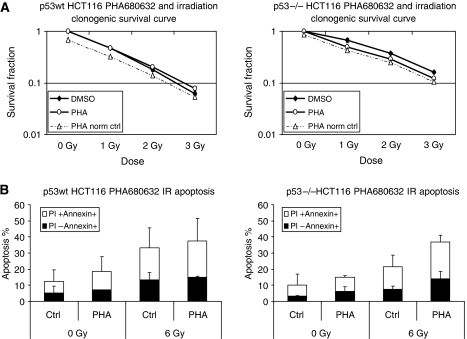
Influence of PHA680632 on the cellular response to irradiation in p53wt *vs* p53−/− HCT116 cells. (**A**) p53-dependent effect of the PHA680632 on clonogenic survival after irradiation; the cells were exposed to 100 nM PHA680632 for 24 h and then irradiated. Data represent the mean of three independent experiments in triplicate, and error bars represent s.d. for p53wt (left) and p53−/− (right) HCT116 cells. The surviving fraction after drug exposure+irradiation is normalised to survival for the same cells treated with the drug alone in the absence of irradiation (plating efficiency: 68.7 and 87%, respectively, for p53wt and p53−/− HCT116 cells exposed to 100 nM PHA680632 alone). For the two HCT116 cell lines, the *P*-values for the clonogenic survival were: *P*<0.0001 for DMSO *vs* PHA norm ctrl, *P*<0.0001 for DMSO *vs* PHA in p53wt HCT116, and *P*=0.0806 for DMSO *vs* PHA in p53−/− HCT116. (**B**) Cytofluorimetric detection of apoptotic parameters in p53 wt (left) and p53−/− (right) HCT116 cells, respectively, exposed to 100 nM for 24 h PHA680832 and then irradiated (6 Gy); 72 h after irradiation, cells were stained with Annexin V and PI and analysed by FACS. Quantification of the data were obtained; error bars represent s.d. For p53−/− HCT116, *P*-value was <0.0001 when comparing PHA680632 alone *vs* IR+PHA680632 and for IR alone *vs* IR+PHA680632 (*P*<0.0001).

Since Aurora-A kinase has been shown to be implicated in p53 degradation, we subsequently explored the radiation response after treatment by PHA680632 (24 h exposure) in cancer cell lines with different p53 functional status. The p53 wild type and p53 knockout HCT116 cells A549 and HT29, another p53 mutant cancer cell line, were chosen. Clonogenic survival assays (colony formation) demonstrated an enhanced radiation response (Figure 3A, ‘PHA norm ctrl’ is survival fraction of a different irradiation dose combined with PHA680632 normalised with ‘control 0 Gy’ that is, cell survival, no irradiation) when the cells were irradiated 24 h after exposure to PHA680632 in p53−/− HCT116 cell line as well as in the p53wt HCT116 cell line.

As shown in Figure 3A, for p53wt HCT116 cell lines, statistic analysis demonstrated that PHA680632 increased the radiation effect (*P*<0.0001), but the effect of PHA680632 tended to reduce as the radiation dose increased. This suggests a slightly sub-additive effect (DER=0.872 at 2 Gy) in HCT116 p53wt cells treated with PHA680632 before irradiation (again, the survival was normalised to the survival curve control, no irradiation). Of interest, as shown in Figure 3A, for p53−/− HCT116 cell lines, statistic analysis demonstrated that PHA680632 increased the radiation effect (*P*<0.0001), that there is an interaction between PHA680632 and IR (*P*=0.0806), and that the effect of PHA680632 increased along with the radiation dose. This suggests rather a supra-additive effect (DER=1.348 at 1 Gy and DER=1.285 at 2 Gy) of irradiation 24 h after exposure to 100 nM PHA680632 in the p53−/− HCT116 cell line. This might reflect a p53 dependency of the effect of PHA680632 on radiation cell killing (Figure 3A). We then conducted an apoptosis assay as defined by Annexin V staining in p53−/− and p53wt HCT116 cells (cells were exposed to 100 nM PHA680832 for 24 h and then exposed to 6 Gy irradiation). As shown in Figure 3B, in p53−/− HCT116 cells, the percentage of apoptotic cells (PI−Annexin+ and PI+Annexin+) was 36.86±4.19 (PHA680632+IR) and 21.54±7.04 (IR alone) (*P*<0.0001), respectively. There was an interaction between PHA680632 and IR, and PHA680632 enhanced radiation-induced apoptosis in p53−/− HCT116 cells. In their p53 wild-type counterparts, p53wt HCT116 cells, the percentage of apoptotic cells, was 37.64±13.96 (PHA680632+IR) and 33.38±12.36 (IR alone), respectively.

**Figure 4:**
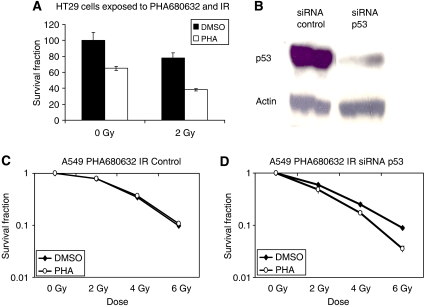
Influence of p53 inhibition by siRNA on the response to PHA680632-IR combination in A549 and HT29 cells. (**A**) Histogram showing the percentage of plating efficiency in HT29 cells treated with 1 *μ*M PHA680632 or DMSO for 24 h; cells were then irradiated to 6 Gy or sham irradiated; error bars represent s.d. The *P*-value was <0.0001 for PHA680632 alone *vs* IR+PHA680632 and IR alone *vs* IR+PHA680632 (*P*<0.0001). (**B**) Western blots showing p53 protein expression 48 h after siRNA p53 or non-specific targeting control siRNA transfection in A549 cells. DMSO *vs* PHA (*P*=0.6444). (**C**, **D**) Clonogenic survival after exposure to 200 nM PHA680632 and IR in siRNA control (left) or siRNA p53 (right) transfected A549 cells. DMSO *vs* PHA (*P*<0.0001).

In the p53 mutant HT29 cell line, irradiation combined with PHA680632 (1 *μ*M PHA680632 24 h exposure before radiation) led to a pronounced inhibition of colony formation as compared with PHA680632 or irradiation alone. This statistical analysis demonstrated that PHA680632 increased the radiation effect (*P*<0.0001) (Figure 4A) (DER=1.205 at 2 Gy).

In the p53wt A549 cell line, a genetic inhibition of p53 by siRNA (Figure 4B) was performed to assess the effect of p53 in response to the combination of PHA680632 and irradiation. We found an increase in radiation response (DER=1.212 at 2 Gy and DER=2.519 at 6 Gy) to the combination of both 200 nM PHA680632 and IR in A549 cells transfected with an siRNA p53 compared to A549 cells transfected with control siRNA and treated with both PHA680632 and IR under the same conditions. There is an interaction between PHA680632 and IR (*P*<0.0001). PHA680632 had little effect (DER=0.994 at 2 Gy) on radiation response in the same cells transfected with control siRNA (*P*=0.6444) (Figure 4C). Thus, this selective Aurora kinases inhibitor, PHA680632, seems to exert a more important influence on the radiation response of cells with a non-functional p53.

### P53 dependency of the influence of inhibition of Aurora-A kinase by siRNA on radiation response in HCT116 cells

**Figure 5:**
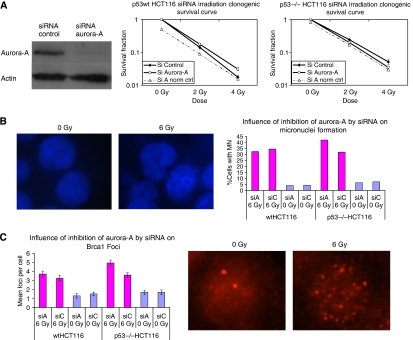
Influence of Aurora-A inhibition by siRNA in response to IR in p53wt *vs* p53−/− HCT116 cells. (**A**) Western blot showing expression of Aurora-A 24 h after siRNA Aurora-A transfection compared with the non-specific targeting siRNA control; clonogenic survival of p53wt (left) or p53−/− (right) HCT116 cells transfected by siRNA Aurora-A or siRNA control; 24 h after transfection, cells were irradiated at indicated doses. Data represent the mean of three independent experiments in triplicate, and error bars represent s.d. (**B** and **C**) Aurora-A inhibition by siRNA influence on IR-induced micronuclei (**B**) and Brca1 foci formation (**C**) in p53wt HCT116 and p53−/− HCT116 cell lines. The percentage of cells with micronuclei (24 h after irradiation) in the p53−/− and p53wt HCT116 cells transfected by siRNA Aurora-A (siA) or siRNA control (siC) and then irradiated to 6 Gy is represented (**B**). There is a significant difference between siA 6 Gy and siC 6 Gy in p53−/− HCT116 (*P*=0.0018), while no difference was found in p53wt HCT116 (*P*=0.5102). No difference has been observed in siA and siC without irradiation (*P*=0.8746 for p53wt HCT116 and *P*=0.614 for p53−/− HCT116). Immunofluorescent images of micronuclei after 6 Gy (or 0 Gy) in p53wt HCT116 cells (**B**). The mean BRCA1 foci (4 h after irradiation) number per cell are shown (**C**) in the p53−/− and p53wt HCT116 cells after transfection by siRNA Aurora-A (siA) or siRNA control (siC) and IR (24 h after transfection of siRNA, 6 Gy). Representative fluorescence microphotographs (Brca1 foci after 6 or 0 Gy) are shown in p53wt HCT116 cells.

To confirm the effect of inhibition of Aurora-A kinase on tumour cells' response to radiation, we used a siRNA approach to inhibit the expression of Aurora-A. The time response of Aurora-A protein inhibition was tested using western blotting. The siRNA selected led to a strong inhibition of Aurora-A expression in the HCT116 cell line 24 h after siRNA transfection (Figure 5A). We then subsequently performed irradiation experiments 24 h after siRNA transfection. In the two HCT116 cell lines, p53−/− and p53wt, a different response to IR after inhibition of Aurora-A was observed (Figure 5A). We observed an increase in radiation cell killing after siRNA Aurora-A transfection when compared to siRNA control in the HCT116 p53−/− cell line (DER=1.213 at 2 Gy and DER=1.454 at 4 Gy) but not in the p53wt HCT116 cell line (DER=0.803 at 2 Gy) (Figure 5A). Indeed, this combination even seemed to exert an antagonistic effect on the p53wt HCT116 cell line. This underscores the role of the p53 functional status in the response after Aurora-A kinase inhibition and irradiation.

We showed previously (Zhang *et al*, 2006) that IR indeed induces the development of micronucleated cells and leads to mitotic catastrophe. This type of cell death occurs during or shortly after a dysregulated or failed mitosis and can be accompanied by morphological alterations, such as micronuclei and multinucleation (Okada and Mak, 2004). Micronuclei can be considered a sign of mitotic catastrophe. We hypothesised that the inhibition of Aurora-A kinase combined with irradiation could induce mitotic catastrophe. Quantification of micronuclei after transfection with siRNA control or Aurora-A showed no significant difference in the number of cells with micronuclei either in the absence (*P*=0.8746) or after IR in the HCT116 wt cell line (*P*=0.5102) (Figure 5B). In the HCT116 p53−/− cell line, siRNA Aurora-A transfection did not significantly affect the number of micronuclei in the absence of irradiation (*P*=0.614), but we observed a significant increase in micronuclei formation after 6 Gy IR compared with siRNA control transfection: 42 *vs* 32% (*P*=0.0018), respectively (Figure 5B). Thus, in the siRNA Aurora-A transfected p53−/− HCT116 cells, there are more cells with micronuclei induced by IR compared with siRNA control transfection; however, this effect was not shown in p53wt HCT116.

It has been reported previously that BRCA1 is phosphorylated at serine-308 by Aurora-A in the centrosome and has been found to correlate with Aurora-A kinase in the G2–M phase transition (Ouchi *et al*, 2004). Regarding the role of BRCA1 in DNA repair, cell cycle checkpoint, and especially the cellular response to IR, we evaluated the influence of Aurora-A inhibition using siRNA on BRCA1 foci formation. The p53−/− or p53wt HCT116 cancer cells were transfected by siRNA Aurora-A or siRNA control for 24 h, then irradiated to 6 or 0 Gy (sham control). At 4 h after IR, the cells were fixed and stained for BRCA1 foci detection. We observed more foci after IR in the nucleus of p53−/− HCT116 cells when Aurora-A expression was inhibited compared to transfection with siRNA control in p53−/− HCT116 cells (4.9 *vs* 3.6); however, in wt HCT116 cells, we could not find any obvious difference between cells transfected by siRNA Aurora-A and control (Figure 5C). This suggests that there is a slight increase in radiation-induced BRCA1 foci after Aurora-A inhibition in p53−/− HCT116 compared to p53wt HCT116 cells.

### *In vivo* experiments in subcutaneous xenograft models using the PHA680632 and IR combination

**Figure 6:**
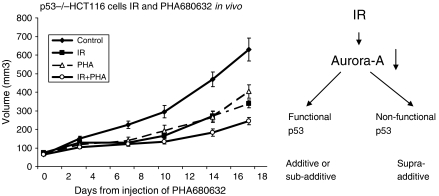
*In vivo* tumour growth delay after PHA680632 and irradiation. Left: *in vivo* experiments in p53−/− HCT116 subcutaneous xenograft. Animals were randomly assigned to control i.p. b.i.d. for 4 days with vehicle (control), PHA-680632 (PHA) 40 mg kg^−1^ alone b.i.d. for 4 days, IR alone 8 Gy in 1 day or a combination of both (IR began just after the second administration of PHA680632); *n*=6 per group, mean tumour volumes±s.e.m. are shown. The respective *P*-values for control *vs* IR+PHA, PHA *vs* IR+PHA and IR *vs* IR+PHA were <0.0001, 0.0003, and 0.0685 respectively. Right diagrammatic representation showing the mechanistic link between Aurora-A, p53, and irradiation.

To explore the radiation response by Aurora-A inhibition *in vivo*, the inhibitor PHA680632 was used in subcutaneous p53−/− HCT116 cell xenograft models. A marked tumour growth delay (TGD) was found in animals treated with PHA680632 alone (40 mg kg^−1^ i.p. b.i.d. for 4 days) compared with the vehicle control. When PHA680632 was combined with IR (one fraction of 8 Gy irradiation) using the same dose of PHA680632, an additive effect on the TGD has been found in p53−/− HCT116 compared with IR alone. *P*-values for PHA680632 *vs* IR+PHA680632 and IR *vs* IR+PHA680632 are 0.0003 and 0.0685, respectively. This suggests that PHA680632 could increase tumour response in combination with irradiation (Figure 6).

## DISCUSSION

### Enhanced radiation-induced cell killing effect *in vitro* by siRNA Aurora-A silencing and a selective Aurora kinase inhibitor, PHA680632

In this study, we analysed the potential effects of Aurora-A kinase inhibition on tumour response to IR using RNAi or a novel selective inhibitor of Aurora kinases, PHA680632, which strongly inhibits the phosphorylation of T288 Aurora-A. It has previously been shown that cells fail to divide after the exposure of PHA680632, yielding polyploid cells leading to a reduction in viability *in vitro* and tumour xenograft regression in mice (Soncini *et al*, 2006). Inhibition of Aurora-A by siRNA Aurora-A or by PHA680632 led to an enhancement of cell killing after exposure to IR in several cell lines *in vitro*. Moreover, we demonstrated that PHA680632 alone could induce a marked tumour growth inhibition *in vivo* and that the combination of PHA680632 and IR could lead to an increased tumour growth inhibition as compared with PHA680632 or IR alone. In our study, low dose PHA680632 did not induce polyploidy while a relatively high dose induced significant polyploidy (data not shown). The PHA680632 concentrations used in our colony formation experiments inhibited very little phospho-histone H3 on serine 10 (data not shown). This might reflect the fact that at low concentrations, PHA680632 potentially exerts more inhibitory effects on Aurora-A than on Aurora-B (IC_50_ on Aurora-A and Aurora-B 27 and 135 nM, respectively). Thus, we chose a relatively low concentration of PHA680632, expecting optimal Aurora-A selectivity.

### P53 influence on response to Aurora-A inhibition by siRNA or PHA680632 combined with irradiation

The combination of both Aurora-A inhibition and radiation led to an increase in the percentage of annexin V-stained cells as well as an increase in micronuclei formation in p53−/− cells when compared to cells exposed to irradiation alone, suggesting that these cells might undergo not only apoptosis but also mitotic catastrophe. An increase in Brca1 foci formation 4 h after irradiation was also observed in p53−/− cells exposed to PHA680632 when compared to cells exposed to irradiation only. Of interest, in the context of a functional p53, exposure to PHA680632 or transfection by siRNA Aurora-A did not increase either the number of apoptotic cells or the number of micronuclei or the Brca1 foci after IR when compared to cells exposed to IR only. This strongly suggests a pivotal role for p53 in response to IR after inhibition of Aurora-A kinase.

Clonogenic survival curves did show the same influence of p53 in response to IR after exposure to PHA680632 or siRNA Aurora-A transfection. However, the impact of this treatment on apoptosis, micronuclei, and Brca1 foci formation after IR did not translate into synergistic enhancement of radiation-induced cell killing. Indeed, we were only able to detect an additive effect of Aurora-A inhibition after Aurora-A inhibition and exposure to IR in p53 non-functional cells using the colony formation survival assays.

G2–M arrest prevents cells from initiating mitosis when they experience DNA damage during G2, or when they progress into G2 with some unrepaired damage (Kastan and Bartek, 2004). p53-independent mechanisms are sufficient to sustain G2 arrest (Kastan and Bartek, 2004) after DNA damage by irradiation. Therefore, interfering with the G2–M checkpoint could be a potential strategy to sensitise cancer cells to ionising radiation (IR). Some authors have suggested that when both checkpoints (such as G1–S and G2–M checkpoint) did not function, cells were more easily sensitised to DNA damaging agents (Zhou and Bartek, 2004). This could be a possible reason for the enhanced radiation response after Aurora-A inhibition in p53-deficient cells.

The integrity of the p53-p21Cip/WAF1-dependent postmitotic checkpoint governs the response to Aurora-A inhibition. It was recently shown that endoreduplication and apoptosis in response to VX-680 are markedly enhanced in cells lacking p53. The difference in response to VX-680 among these cell lines correlates with the timing of induction of p21Cip/WAF1 and its ability to inhibit cyclin E-cdk2 activity (Gizatullin *et al*, 2006). These data also suggest the interplay between p53, its downstream effectors, and Aurora-A.

PHA680632 brought about additive interaction with radiation in terms of induced cell death in p53 non-functional cells. Such additivity may be beneficial in chemo-radiotherapeutic combinations. Indeed, supra-additive interaction may lead to acute hypertoxicity, reduction of the maximum tolerated doses of both drug and radiation and treatment failure. Contrary to a widely held opinion, radiosensitisation should be considered with care when it comes to eliciting inhibition of radiation recovery (Balosso *et al*, 1995). Well-known examples of limiting toxicities are adriamycin (Mayer *et al*, 1976) and bleomycin (Peters *et al*, 1988).

It should be recalled here that among the most useful mechanisms in chemo-radiotherapeutic combinations are spatial cooperation, reoxygenation, and inhibition of tumour repopulation. Reduction in tumour volume after chemotherapy, when it occurs, may result in improved blood supply to the tumour, leading to reoxygenation and increased radiosensitivity (Hennequin and Favaudon, 2002). PHA680632 and radiotherapy might be used concomitantly or in close temporal proximity, potentially without acute or late healthy tissue complications.

In conclusion, we demonstrate that Aurora-A inhibition by the Aurora kinase inhibitor, PHA680632, in association with radiation leads to an additive effect in cancer cells, especially in p53-deficient cells but does not act as a radiosensitiser *in vitro* or *in vivo*. However, further experiments are needed for a better understanding of the interplay between the molecular checkpoints that are activated by IR and the consequences of Aurora kinases inhibition. The results suggest the importance of Aurora-A kinase targeting in combination with IR.
